# Characterization of a factor VIII/immunoglobulin heavy chain μ double-knockout mouse model of hemophilia A for long-term exposure to factor VIII proteins

**DOI:** 10.1016/j.rpth.2025.103277

**Published:** 2025-12-02

**Authors:** Lara Monica, Olga Oleshko, Lina Aires, Silvio Wuschko, Jonas Füner, Helmut Paul, Paul Schauerte, Ditte Starberg Jespersen, Larisa Belyanskaya, Andreas Tiede, Sonja Werwitzke

**Affiliations:** 1 Hematology, Hemostasis, Oncology, and Stem Cell Transplantation, Hannover Medical School, Hannover, Germany; 2 preclinics Gesellschaft für präklinische Forschung mbH, Potsdam, Germany; 3 Octapharma AG, Lachen, Switzerland; 4 Octapharma Pharmazeutika Produktionsges mbH, Vienna, Austria; 5 Taconic Biosciences GmbH, Leverkusen, Germany; 6 Taconic Biosciences A/S, Lille Skensved, Denmark; 7 Institute of Clinical Chemistry and Central Laboratory, Hannover Medical School, Hannover, Germany

**Keywords:** FVIII inhibitors, FVIII pharmacokinetics, FVIII-specific B- and T-cell activation, hemophilia A, mouse model

## Abstract

**Background:**

Deficiency of coagulation factor (F)VIII is the key characteristic of hemophilia A. The FVIII knockout mouse model is a valuable tool for investigating disease mechanisms and evaluating the pharmacokinetics (PK) and efficacy of therapeutic agents. However, its utility for long-term studies, particularly those focused on prophylaxis, is limited by the development of anti-FVIII antibodies following repeated FVIII administration.

**Objectives:**

To develop a FVIII knockout model that does not generate antibodies against FVIII but is not severely immunosuppressed.

**Methods:**

Established FVIII single-knockout (FVIII^-/-^; SKO) mice were crossed with the immunoglobulin heavy chain μ (Ighm) knockout strain to generate FVIII/Ighm double-knockout (FVIII^-/-^/Ighm^-/-^; DKO) mice. Both SKO and DKO mice received 4 intravenous doses of recombinant human FVIII on days 0, 7, 14, and 21. Peripheral blood, bone marrow, and spleen samples were collected to assess (i) the immune response to FVIII, (ii) FVIII PK after repeated dosing, and (iii) overall immune status.

**Results:**

DKO mice did not develop detectable anti-FVIII antibodies. The formation of FVIII-specific antibody-secreting cells was abrogated, and the presence of FVIII-specific T cells was substantially reduced. PK analysis performed after 3 weeks of FVIII exposure showed variable reductions in FVIII recovery and half-life in SKO mice. In contrast, PK parameters in DKO mice remained consistently within the expected physiological range.

**Conclusion:**

The DKO mouse model can be used for long-term studies of FVIII products and other hemostatic proteins, enabling the evaluation of repeated-dose PK and prophylactic efficacy without interference from inhibitor formation.

## Essentials

• The hemophilia A mouse model is not suitable for prophylaxis studies due to inhibitor formation.
• We developed a new model lacking the immunoglobulin heavy chain μ.
• After 3 weeks of exposure, mice did not develop inhibitors and had stable pharmacokinetics.
• This mouse model can be used to study long-term factor prophylaxis.

## 1 Introduction

Hemophilia A (HA) is an X-linked disorder characterized by a deficiency of coagulation factor (F)VIII, a cofactor of activated FIX in blood coagulation [1], resulting in a lifelong tendency to bleed. Repeated bleeding, especially into the large joints, predominantly the ankles, knees, and elbows, causes chronic inflammation that ultimately results in hemophilic arthropathy [2]. Bleeding events also occur in soft tissues, mucous membranes, or the central nervous system and can be life-threatening [3]. Osteoporosis is common in people with HA, though the underlying cause remains poorly understood [4]. Joint bleeding, a key complication contributing to musculoskeletal deterioration, can be largely prevented through prophylactic administration of current hemophilia treatments, including plasma-derived FVIII concentrates, standard recombinant FVIII (rFVIII), and extended half-life rFVIII. Despite the clinical success of these products in reducing bleeding risk, not all patients are protected from hemophilic arthropathy [[5], [6], [7]]. The need for frequent factor administration, usually 1 to 3 times per week, poses challenges, and periods of low FVIII activity levels during the prophylactic cycle can leave patients vulnerable to breakthrough bleeds. Furthermore, 30% to 40% of patients with severe HA develop neutralizing immunoglobulin (Ig)G antibodies, known as inhibitors, against the administered FVIII, leading to treatment failure [8].

Nonfactor replacement therapies have recently emerged as a promising approach to overcoming challenges in HA management [9,10]. These include: (i) the bispecific monoclonal antibody emicizumab, which mimics activated FVIII by bridging activated FIX and FX [11,12]; (ii) monoclonal antibodies, such as concizumab [13] or marstacimab [14], which inhibit tissue factor pathway inhibitor; and (iii) the small interfering RNA fitusiran [15], which suppresses antithrombin production to rebalance coagulation in HA. These therapies are administered subcutaneously and have longer half-lives. However, clinical studies have reported a variable risk of breakthrough bleeding. Additionally, emerging preclinical data suggest that FVIII may play roles beyond coagulation, particularly in maintaining vascular endothelial integrity [16] and in trabecular bone remodeling [4,17,18], underscoring the need for further research to optimize prophylactic treatments and improve long-term outcomes in HA.

Animal models are important in the early stages of new drug development and for evaluating how treatments interact with key disease pathways, such as those involved in HA-related joint pathology [19]. The well-known FVIII-deficient mouse model [20,21] is used to evaluate the pharmacokinetics (PK) and pharmacodynamics (PD) of investigational drugs in preclinical studies [[22], [23], [24]]. A major limitation of this model is the development of an immune response to repeatedly administered human FVIII products [[25], [26], [27]]. Such antidrug antibodies (ADAs) can significantly affect the PK and PD of new therapeutic substances. Consequently, PK studies of the FVIII single-knockout (FVIII^-/-^; SKO) mouse model were mostly limited to single-dose administration, and long-term efficacy data for this model are lacking [22,28,29]. To address this problem, severe immunodeficient mice lacking FVIII and CD4, or Non-Obese Diabetic/Severe Combined Immunodeficient (NOD/SCID) mice, were used in preclinical gene therapy studies [[30], [31], [32]]. These models successfully allowed for FVIII expression, but the severe combined immunodeficiency caused by the complete absence of T helper cells in the FVIII/CD4 knockout model and the complete absence of T and B cells in the NOD/SCID model might represent a significant limitation. The absence of a functional T-cell compartment in these models may affect aspects of inflammatory responses and the progression of joint disease and arthropathy after bleeding episodes. Likewise, handling a mouse strain with severe combined immunodeficiency is challenging for laboratories and for animal welfare.

Our goal was to generate a new HA mouse model that is incapable of developing ADAs in response to FVIII administration but is not severely immunosuppressed. The objective was to establish a preclinical model suitable for long-term treatment studies, aimed at advancing our understanding of the pathophysiological mechanisms underlying hemophilic joint disease and facilitating the development of effective preventive strategies.

For this purpose, we crossed the SKO mouse model with the Ig heavy chain μ (Ighm) knockout (Ighm^-/-^) strain [33], which is unable to generate mature B cells and produce subsequent antibodies. Although the Ighm^-/-^ strain lacks an important part of adaptive immunity, it is not generally immunocompromised. It was shown that the absence of B cells and their associated antigen-presenting mechanisms in Ighm-deficient mice had a minor effect on B-cell-independent T-cell responsiveness [34]. The FVIII/Ighm double-knockout (FVIII^-/-^/Ighm^-/-^; DKO) strain should not be able to mount ADAs after repeated FVIII exposure.

This study was designed as a proof-of-concept to demonstrate that long-term studies of repetitive FVIII administrations are feasible in the newly generated DKO mouse model, given the lack of a FVIII-specific B-cell response. As expected, antibody formation was observed in the SKO mice, resulting in reduced FVIII recovery and half-life. In contrast, this was not observed in DKO mice. The lack of mature B cells in DKO mice was confirmed by comparing the spleen and bone marrow (BM) B- and T-cell compartments with those of SKO mice.

## 2 Methods

### 2.1 Proteins and peptides

Recombinant human FVIII (rhFVIII; Simoctocog alfa, Octapharma AG) was produced in human embryonic kidney (HEK)293F cells [35] and supplied as lyophilized powder by Octapharma Pharmazeutika Produktionsges mbH. It was reconstituted in water for injections under aseptic conditions. Specific peptide sequences within the full-length FVIII sequence (UniProt ID: P00451) that may potentially be recognized by T cells as epitopes were selected using NetMHCII 2.3 (Morten Nielsen, Ole Lund, and collaborators), as described in Table 1. Custom-synthesized FVIII-derived peptides (ProteoGenix) were reconstituted in water (or in a water/acetonitrile mixture when specified) at a final concentration of 1 mg/mL, mixed in equal parts, and stored at −20 °C until use.

**Table 1:** Factor VIII peptides.

Position	Sequence	FVIII domain	Purity, %
53	VPKSFPFNTSVVYKK	A1	91.79
622	LEDPEFQASNIMHSI	A2	88.20
1723	ERLWDYGMSSSPHVL	A3	93.89
1409	IAKVSSFPSIRPIYL	B	80.03
2154	IKHNIFNPPIIARYI	C1	82.71
2216	TNMFATWSPSKARLH	C2	90.84

A total of 6 FVIII peptides, predicted to have a strong interaction with major histocompatibility complex class II molecules specific for the experimental mouse strain (haplotype H-2IA-b), were selected.

FVIII, factor VIII.

### 2.2 Mice, husbandry, and immunization protocol

A novel DKO mouse model was generated by crossing FVIII-deficient mice (B6;129S4-F8^tm2Kaz^) [21] with the Ighm^-/-^ model (B6.129S2/Ighm^tm1Cgn^/J) [33], which is unable to generate antibodies due to the absence of mature B cells. DNA for genotyping by polymerase chain reaction amplification was isolated from stool pellets of offspring or from ear biopsies by the breeder at setup to allow the assumption of offspring genotypes. Offspring genotyping was performed by standard polymerase chain reaction, as previously described in detail for FVIII knockout, and according to the Jackson Laboratory protocols for Ighm [36].

For the crossbreeding program, FVIII-deficient mice were mated to mice with homozygous targeted disruption of the Ighm gene. In the F2 generation, offspring were screened for homozygous Ighm gene disruption and hemizygous (males) or homozygous (females) FVIII gene disruption.

The DKO mice were provided by Taconic Biosciences A/S, and the SKO mice were purchased from Jackson Laboratory (B6;129S-F8^tm1Kaz^/J). All animal experiments were conducted at preclinics GmbH.

Mice were housed individually in MII cages with unlimited access to food and water, a 12-hour/12-hour light-dark cycle, and an air temperature between 20 and 23 °C. Each animal was individually identified (Identifier [ID] 1-36). All animal experiments complied with the Animal Research: Reporting of In Vivo Experiments (ARRIVE) guidelines [37] and the European Union Directive 2010/63/EU for animal experiments [38], and were approved by the relevant authorities.

Mice were enrolled in experiments at 11-13 weeks of age and were randomized into 6 experimental groups stratified by sex (Table 2). RhFVIII was administered via tail vein injection at a dose of 6 International Units (IU) per mouse (approximately 200 IU/kg body weight) on days 0, 7, 14, and 21 (groups 1, 2, 4, and 5, respectively), or left untreated (groups 3 and 6). Mice were sacrificed on day 21 (groups 1 and 4 exactly 1 hour after the last dose of rhFVIII; groups 2 and 5 exactly 14 hours after the last dose of rhFVIII).

**Table 2:** Experimental groups.

Study features	Group 1 (*n* = 6)	Group 2 (*n* = 6)	Group 3 (*n* = 6)	Group 4 (*n* = 6)	Group 5 (*n* = 6)	Group 6 (*n* = 6)
Strain	SKO (FVIII^-/-^)	SKO (FVIII^-/-^)	SKO (FVIII^-/-^)	DKO (FVIII^-/-^/Ighm^-/-^)	DKO (FVIII^-/-^/Ighm^-/-^)	DKO (FVIII^-/-^/Ighm^-/-^)
Sex, male/female	3/3	3/3	3/3	3/3	3/3	3/3
rhFVIII dose, IU/mouse	6	6	-	6	6	-
Time of blood sampling	D21 1 h after the fourth rhFVIII injection	D21 14 h after the fourth rhFVIII injection	D21	D21 1 h after the fourth rhFVIII injection	D21 14 h after the fourth rhFVIII injection	D21

D21, day 21; DKO (FVIII^-/-^/Ighm^-/-^), FVIII/Ighm double-knockout strain; FVIII, factor VIII; IU, International Units; rhFVIII, recombinant human factor VIII; SKO (FVIII^-/-^), FVIII single-knockout strain.

### 2.3 Blood sampling and blood assays

Blood was collected after anesthesia via the retro-orbital plexus using nonheparinized End-to-End Minicaps (25 μL, Hirschmann Laborgeräte GmbH & Co KG) into tubes containing sodium citrate (1/10 volume, 3.2 mmol/L). Plasma was obtained by immediate centrifugation of blood samples at 4 °C for 10 minutes at 5000 × *g*. The plasma was aliquoted into 0.5 mL microtubes and stored at −70 °C until analysis. Anti-FVIII antibody titers in murine plasma samples were measured by enzyme-linked immunosorbent assay (ELISA) as previously described [27]. Quantitative assessment of rhFVIII levels was performed using the Human FVIII ELISA Kit (total FVIII antigen, Abcam) according to the manufacturer’s instructions with minor modifications. Briefly, plasma samples were diluted 1:8 in assay blocking buffer, and 100 μL of the diluted sample was added to each well of the precoated 96-well plate. Plates were incubated for 30 minutes at room temperature (RT) with shaking at 300 rpm. After a wash step, 100 μL of the primary antibodies was added and incubated under the same conditions. After additional washes, 100 μL of streptavidin-horseradish peroxidase conjugate was added and incubated for another 30 minutes at RT with shaking at 300 rpm. Following a final wash cycle, 100 μL of TMB substrate was added for 10 minutes. The reaction was stopped by adding 50 μL of H_2_SO_4_, and the absorbance was measured at 450 nm using a Multiskan Ascent ELISA Reader (Thermo Fisher Scientific). A standard calibration curve was prepared using the same rhFVIII product used for injections. Both the experimental samples and the standards contained the same proportion of murine plasma and were processed in parallel.

### 2.4 BM and splenocytes isolation

BM cells were harvested by flushing dissected femurs with washing buffer (phosphate-buffered saline [PBS] + 1% heat-inactivated [HI] FBS). The resulting cell suspension was filtered through a 70-μm cell strainer to obtain a single-cell suspension and stored at −70 °C in Bambanker cryopreservation solution (Nippon Genetics) until further use. Splenocytes were isolated from dissected spleens by mechanical dissociation using a gentleMACS dissociator (Miltenyi Biotec). Cell suspensions were washed in washing buffer (PBS + 1% HI FBS) and subjected to red blood cell lysis using an in-house prepared ammonium chloride-based lysis buffer. The suspension was then filtered through a 40-μm strainer to ensure a single-cell population. Freshly isolated splenocytes were used for enzyme-linked immunospot (ELISpot) assays. For flow cytometric analyses (FACS), splenocytes were cryopreserved in Bambanker solution and stored at −70 °C for up to 2 weeks prior to staining.

### 2.5 B-cell assay

Washed splenocytes from individual mice were pooled according to their experimental group and seeded into 96-well clear, round-bottom cell culture plates (Corning Inc). Cells were cultured in RPMI medium (Corning) (1% penicillin/streptomycin [Pen/Strep] and 10% HI FBS) supplemented with rhFVIII (1 or 3 IU/mL) or medium alone (unstimulated controls). For each condition, 2 cell densities were tested: 4 × 10^5^ and 6 × 10^5^ cells/well. After 6 days of incubation at 37 °C and 5% CO_2_, cells were subjected to ELISpot assays as described [27]. Briefly, Multiscreen-IP MAIPS4510 ELISpot Plates (Merck/Millipore) were preactivated with 35% ethanol and coated with rhFVIII (2 IU/well) overnight at 4 °C. Plates were blocked with RPMI (1% Pen/Strep and 10% HI FBS) prior to cell seeding. Cultured splenocytes were transferred to the coated wells and incubated at 37 °C and 5% CO_2_ for 5 hours. After 3 washing steps with Dulbecco's Phosphate Buffered Saline (DPBS) and DPBS + 0.1% Tween, HRP-conjugated donkey anti-mouse IgG antibody (Chemicon; dilution 1:1500 in PBS) was added, and plates were incubated overnight at 4 °C. Spots were developed using AEC Substrate (BD Biosciences) and quantified using an automated ELISpot Reader and software (A.EL.VIS). As a baseline control, freshly isolated splenocytes were analyzed by ELISpot without any rhFVIII *in vitro* restimulation. These samples were tested at the same 2 cell densities (4 × 10^5^ and 6 × 10^5^ cells/well) using the same protocol [27].

### 2.6 T-cell assay

Washed splenocytes from individual mice were pooled according to their experimental group, resuspended in RPMI medium (1% Pen/Strep and 10% HI FBS), and analyzed using the ELISpot Plus: Mouse IFN-γ (ALP) Kit (3321-4AST, Mabtech) for the detection of murine cells secreting interferon (IFN)-γ. A total of 2 × 10^5^ cells/well were seeded into the precoated IFN-γ ELISpot plates and stimulated *in vitro* with either rhFVIII (1 or 3 IU/mL) or a pool of selected FVIII-derived peptides (Table 1), or cultured with medium alone as a negative control. Additionally, a pool of splenocyte samples from all experimental groups was stimulated with concanavalin A (2.5 μg/mL) as a positive control in both experiments. After incubation at 37 °C and 5% CO_2_ for either 15 or 39 hours, cells were washed 5 times with PBS and incubated for 2 hours at RT with a biotinylated anti-mouse IFN-γ detection antibody (R4-6A2-biotin [part of the ELISPOT kit from Mabtech], 1 μg/mL in PBS + 0.5% HI FBS). After 5 additional PBS washes, streptavidin-ALP secondary antibody (part of the ELISPOT kit from Mabtech) was added at a 1:1000 dilution (PBS + 0.5% HI FBS) and incubated for 1 hour at RT. Following the final wash steps, cytokine-secreting cells were visualized using the ready-to-use BCIP/NBT-plus substrate solution. Spot development was performed according to the manufacturer’s instructions, and quantification was carried out using an automated ASTOR ELISpot reader (Mabtech).

### 2.7 Flow cytometry

Flow cytometry was performed according to standard protocols using a MACSQuant Analyzer 10 (Miltenyi Biotec). Frozen splenocytes or BM cells were isolated from individual mice (*n* = 6 per group, 3 females and 3 males; see Table 2), thawed at 37 °C, washed, and resuspended in MACS Rinsing Solution (Miltenyi Biotec). Staining and washing steps were performed in 96-well clear, round-bottom, nontreated polypropylene microplates (Corning Inc). Cells were plated and stained with REAfinity recombinant antibodies for flow cytometry (all from Miltenyi Biotec). Viability was assessed using Viobility 405/520 Fixable Dye (Miltenyi Biotec). For immunotyping of B- and T-cell populations, splenocytes and BM cells were stained with the following antibodies: B cells: CD45-VioBlue, CD19-PE, and IgM-VioBright B515; T cells: CD45-PE-Vio615, CD3-APC, CD4-PE-Vio770, CD8-VioBright B515, CD44-APC-Vio770, and CD62L-VioBright V423. Briefly, cells were incubated with the viability dye (Viobility 405/520; diluted 1:100 in 1× PBS) followed by the respective antibody panels (B- or T-cell markers) for 20 minutes in the dark at RT. After 3 washing steps, cells were resuspended in PBS for analysis. Data were analyzed using FlowJo version 10.9.0 (FlowJo LLC). Gate settings were established using unstained controls and single-stained compensation controls. All populations were gated on single, viable leukocytes. The definitions of the lymphocyte subpopulations are summarized in Table 3.

**Table 3:** Cell surface markers used for the characterization of immune cell types.

Cell type	Characterization				
Pre-B cells	CD45+	CD19+	IgM−		
B cells	CD45+	CD19+	IgM+		
CD8 cytotoxic T cells	CD45+	CD3+	CD8+		
CD4 T helper cells	CD45+	CD3+	CD4+		
CD4 TCM cells [39]	CD45+	CD3+	CD4+	CD44+	CD62L+
CD4 TEM cells [39]	CD45+	CD3+	CD4+	CD44+	CD62L−
CD4 T naive cells	CD45+	CD3+	CD4+	CD44−	CD62L+

CD, cluster of differentiation; Ig, immunoglobulin; TCM, T central memory cells; TEM, T effector memory cells.

### 2.8 Statistical analysis

Data were aggregated and reported as means (SDs), medians (IQRs), and numbers (%), as appropriate. GraphPad Prism version 10 (GraphPad Software), R version 4.3.0 (R Foundation for Statistical Computing), and FlowJo version 10.9.0 (FlowJo LLC) were used for statistical analysis, graphical illustration, and plotting.

## 3 Results

### 3.1 DKO mice do not generate antibodies against injected rhFVIII

**Figure 1:**
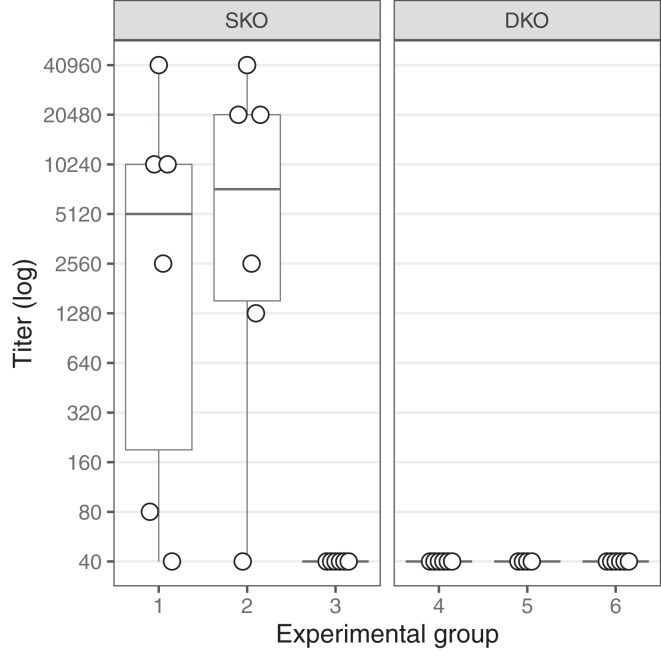
Anti-factor (F)VIII antibody titers after repetitive recombinant human FVIII (rhFVIII) administration in FVIII single-knockout (SKO) and FVIII/Ighm double-knockout (DKO) mouse models. Anti-FVIII antibody titers were measured by enzyme-linked immunosorbent assay in murine plasma samples on day 21 in rhFVIII-treated SKO mice (groups 1 and 2), rhFVIII-treated DKO mice (groups 4 and 5), and untreated SKO (group 3) and DKO (group 6) mice. Samples were collected 1 hour (in groups 1 and 4) or 14 hours (in groups 2 and 5) after the fourth rhFVIII injection (see Table 2). The box and whisker plot displays the median and IQR for each group (*n* = 6), with each dot representing the results of an individual mouse.

Anti-FVIII antibody formation was not detected in any DKO mice following repeated rhFVIII administration. In contrast, as expected, SKO mice injected with rhFVIII developed variable but predominantly high levels of antibody titers (Figure 1). The antibody titers in SKO mice were notably higher in the group sampled 14 hours after the fourth rhFVIII injection compared with the group sampled at 1 hour, indicating ongoing differentiation of antibody-secreting cells (ASCs) at the later time point. A correlation was observed between anti-FVIII antibody formation and circulating rhFVIII levels in the plasma of SKO mice (data not shown). Specifically, mice with high antibody titers (>1:80) had either undetectable or markedly reduced rhFVIII plasma concentrations (below 1 IU/mL). In contrast, the 2 SKO mice with low antibody titers (<1:80) maintained measurable rhFVIII levels above 1 IU/mL.

### 3.2 FVIII PK parameters

**Figure 2:**
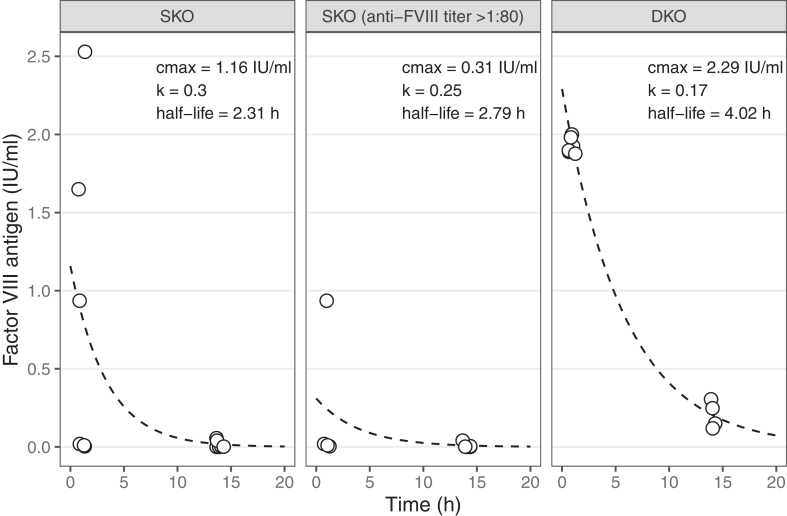
Factor (F)VIII pharmacokinetics after repetitive recombinant human FVIII administration in FVIII single-knockout (SKO) and FVIII/Ighm double-knockout (DKO) mouse models. Recombinant human FVIII concentration (IU/mL) is shown as a function of time. The FVIII antigen concentration was measured, and pharmacokinetic parameters were estimated using a noncompartment model curve and are displayed in the figure. C_max_, maximum plasma concentration; IU, International Units; k, elimination rate constant.

After quantifying injected rhFVIII in plasma at 1 and 14 hours after the fourth rhFVIII administration on day 21 in both mouse strains, a PK curve was generated, and PK parameters were calculated using noncompartmental analysis, as shown in Figure 2. The maximum plasma concentration for both strains was observed at the 1-hour postinjection time point after the fourth rhFVIII administration. The average maximum FVIII plasma concentration in DKO mice (2.29 IU/mL) was notably higher than in SKO mice (1.16 IU/mL), indicating a more efficient initial rise in circulating FVIII levels in the absence of neutralizing antibodies. The half-life of rhFVIII was also longer in DKO mice (4.02 hours) than in SKO mice (2.31 hours), with a corresponding reduction in the elimination rate constant in DKO mice (0.17 vs 0.30). The slower clearance of FVIII in the DKO mice was most likely attributable to a lack of antibody-mediated elimination. The SKO mice showed greater interindividual variability in plasma rhFVIII levels, owing to variable anti-FVIII antibody levels. Specifically, mice with high inhibitor titers (>1:80) displayed significantly reduced plasma rhFVIII concentrations (<1 IU/mL), which precluded the generation of a reliable PK curve in these animals.

### 3.3 FVIII-specific B-cell response

**Figure 3:**
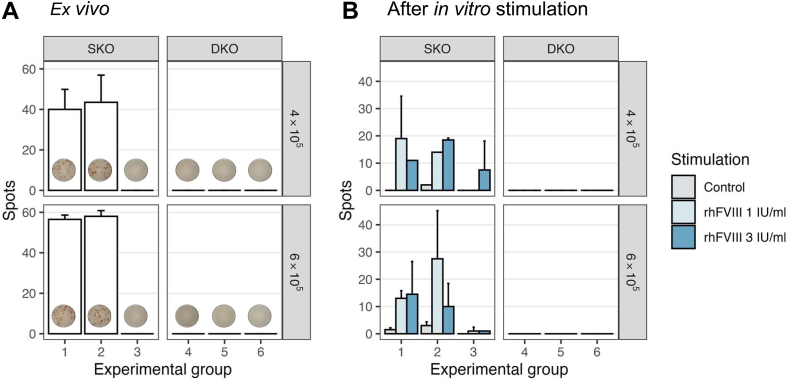
Factor (F)VIII-specific antibody-secreting cells after repetitive recombinant human FVIII (rhFVIII) administration in FVIII single-knockout (SKO) and FVIII/Ighm double-knockout (DKO) mouse models. Splenocytes from mice in each individual study group (groups 1 and 2: rhFVIII-treated SKO mice, sampled on day 21, 1 and 14 hours after the fourth rhFVIII injection, respectively; group 3: untreated SKO mice; groups 4 and 5: rhFVIII-treated DKO mice, sampled on day 21, 1 and 14 hours after the fourth rhFVIII injection, respectively; and group 6: untreated DKO mice) were pooled for analyses and tested in technical duplicates for FVIII-specific antibody-secreting cells by enzyme-linked immunospot (A) *ex vivo* on day 0, directly after splenocyte isolation, or (B) after 6 days of *in vitro* restimulation with different concentrations of rhFVIII (0-3 IU/mL). Grouped bars show the number of detected spots. Each test was performed twice using 4 × 10^5^ or 6 × 10^5^ cells/well. The mean ± SD is shown.

In line with the observed antibody results, pooled splenocytes from FVIII-treated SKO mice (groups 1 and 2) showed the presence of FVIII-specific ASCs immediately upon isolation (Figure 3A).

In contrast, no FVIII-specific ASCs were detected in either FVIII-treated or untreated DKO mice (groups 4-6) at the same time point, demonstrating a complete absence of an ongoing FVIII-specific B-cell response. To further assess the presence and function of memory B cells, splenocytes were cultured *in vitro* for 6 days with rhFVIII (1 or 3 IU/mL) using an established cell culture system [40,41]. Upon rhFVIII restimulation, SKO splenocytes showed robust differentiation of FVIII-specific ASCs, confirming the presence of FVIII-specific memory B cells (Figure 3B). In contrast, no such response was observed in any DKO mouse, confirming the absence of both FVIII-specific plasma cells and memory B-cell populations in these mice. These results strongly support the conclusion that DKO mice are functionally deficient in FVIII-specific B-cell responses, enabling their use in long-term preclinical studies without the confounding effects of inhibitor formation.

### 3.4 FVIII-specific T-cell response

**Figure 4:**
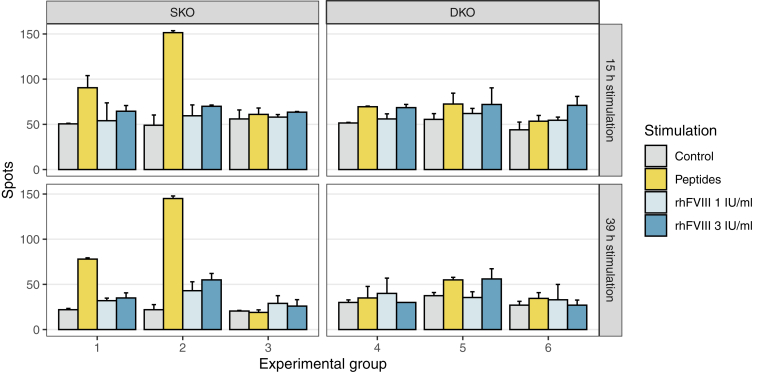
Factor (F)VIII-specific T cells after repetitive recombinant human FVIII (rhFVIII) administration in FVIII single-knockout (SKO**)** and FVIII/Ighm double-knockout (DKO**)** mouse models. Splenocytes from mice in each individual study group (groups 1 and 2: rhFVIII-treated SKO mice, sampled on day 21, 1 and 14 hours after the fourth rhFVIII injection, respectively; group 3: untreated SKO mice; groups 4 and 5: rhFVIII-treated DKO mice, sampled on day 21, 1 and 14 hours after the fourth rhFVIII injection, respectively; and group 6: untreated DKO mice) were pooled for analyses and tested in technical duplicates for interferon-γ by enzyme-linked immunospot. Splenocytes (2 × 10^5^ cells/well) were stimulated in duplicate with rhFVIII (1 or 3 IU/mL) or FVIII peptides (10 μg/mL) for 15 or 39 hours. Bars represent the number of interferon-γ-secreting cells. Unstimulated cells served as controls. The mean ± SD is shown.

To assess FVIII-specific T-cell activation, pooled splenocytes from each experimental group were stimulated *in vitro* with either (i) intact rhFVIII or (ii) a pool of rhFVIII-derived peptides. T-cell responses were assessed using an IFN-γ ELISpot assay (Figure 4). Six peptides representing potential CD4^+^ T-cell epitopes were selected using the NetMHCII 2.3 prediction algorithm based on their predicted strong binding (rank < 2) to H-2IA-b class II molecules (Table 1). These peptides span distinct regions of the FVIII protein and were chosen to enable robust restimulation of FVIII-reactive T cells *in vitro*. Stimulation with intact rhFVIII induced only a modest increase in IFN-γ secretion, suggesting limited T-cell activation under these conditions. In contrast, peptide-based stimulation resulted in efficient *in vitro* restimulation, highlighting more efficient presentation and recognition of processed epitopes. In particular, splenocytes from SKO mice treated *in vivo* with rhFVIII (groups 1 and 2) showed robust IFN-γ responses upon peptide stimulation, indicative of a primed FVIII-specific CD4^+^ T-cell population. In contrast, DKO mice showed minimal IFN-γ production in response to both rhFVIII and peptide pools, suggesting that the lack of ASCs and memory B cells in the DKO strain may also compromise antigen-specific T-cell priming, likely due to disrupted B-cell-mediated antigen presentation. Overall, these results demonstrate a marked attenuation of FVIII-specific T-cell responses in DKO mice, further validating the model's utility for evaluating FVIII therapeutics in the absence of an adaptive immune response.

### 3.5 T and B cells in the SKO and DKO mouse models

**Figure 5:**
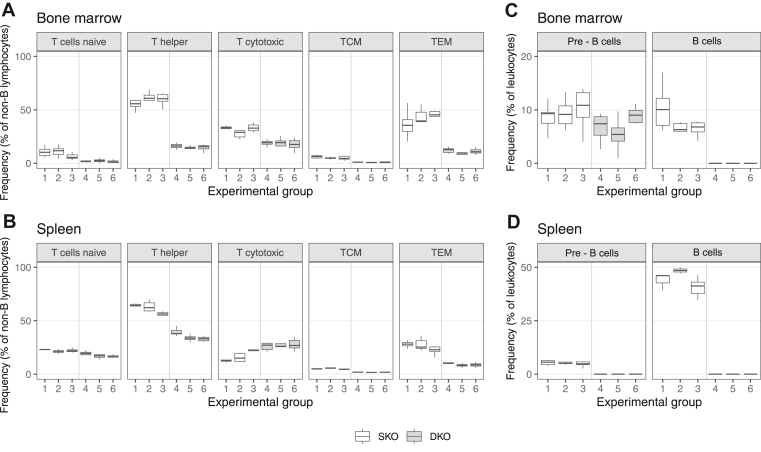
Spleen and bone marrow B-cell profile (% of leukocytes) and T-cell profile (% of non-B lymphocytes). Flow cytometric analysis of B- and T-cell populations in factor (F)VIII single-knockout (SKO; groups 1-3) and FVIII/Ighm double-knockout (DKO; groups 4-6) strains across different treatment conditions (see Table 2) with equal sex distribution. (A, C) Bone marrow cells and (B, D) splenocytes were stained with CD45 and Viobility 405/520 Fixable Dye to gate living leukocytes. Further analysis of (C) bone marrow and (D) spleen cells involved CD19 and immunoglobulin M staining to distinguish pre-B and B cells. T-cell populations in the (A) bone marrow and (B) spleen were characterized using CD3 and CD8 (cytotoxic T cells), CD3 and CD4 (T helper cells), and CD3, CD4, CD44, and CD62L (T central memory [TCM] cells, T effector memory [TEM] cells, and naive T cells). The percentage of each population was quantified. Median, upper, and lower quartiles for each group (*n* = 6) are shown in the box and whisker plot.

T-cell frequencies in the BM and spleen were quantified by FACS and expressed as a relative percentage of non-B viable, singlet leukocytes to allow direct comparison between the SKO strain (groups 1-3) and the DKO strain (groups 4-6; Figure 5A, B). Overall, SKO mice exhibited higher total T-cell frequencies compared with their DKO counterparts, with the exception of splenic cytotoxic T cells, which were similar between the strains. No notable differences were found between male and female mice or between treatment groups within the same strain.

Since mammalian B lymphocytes originate in the BM and mature in secondary lymphoid tissues such as the spleen [42], both BM and splenic B-cell populations were analyzed in parallel (Figure 5C, D). Immature B cells (defined as pre-B cells; CD19^+^ and IgM^-^) were detected in the BM of both SKO and DKO strains, but were present only in the spleen of the SKO strain. Mature B cells (CD19^+^ and IgM^+^), indicative of fully developed B lymphocytes, were identified in both the BM and spleen of SKO mice only. The absence of mature B cells in DKO mice reflects the targeted disruption of the Ighm gene, which is essential for the expression of membrane-bound IgM and the development of functional B-cell receptors. As functional B lymphocytes are defined by their capacity to express antigen-specific Ig receptors [42], their complete absence in DKO animals confirms the immunophenotypic integrity of the model.

## 4 Discussion

In this study, we established a novel HA mouse model, the DKO, characterized by a combined deficiency in FVIII and Ig production, rendering the mice incapable of producing anti-FVIII antibodies in response to repeated FVIII administration.

To evaluate the model's suitability for long-term PK studies, we performed a 21-day PK study in which we administered weekly injections of rhFVIII in both SKO and DKO mice. As expected, SKO mice developed variable, but often high anti-FVIII antibody titers following repeated FVIII exposure, which significantly impacted FVIII recovery and half-life. In contrast, DKO mice exhibited consistent FVIII PK, underscoring the utility of the DKO model for longitudinal FVIII studies without the confounding effects of inhibitor formation. FACS confirmed the complete absence of B cells in both the BM and spleen of DKO mice. More specifically, the FVIII-specific B-cell ELISpot assay confirmed the abrogation of FVIII-specific ASCs in DKO mice, even after *in vitro* restimulation, confirming the functional abrogation of the FVIII-specific B-cell response. Notably, CD8^+^ cytotoxic T-cell levels were comparable between SKO and DKO strains, while CD4^+^ T cells were modestly reduced in DKO mice. These results are consistent with previous findings [43] showing that CD8^+^ T cells can develop and be maintained independently of B cells and their antigen-presenting functions, whereas CD4^+^ memory T-cell maintenance is at least partially dependent on B-cell-mediated antigen presentation. Even if these results seemed to be directly linked to a lack of B cells, we cannot completely exclude the possibility that the differences noted in the T-cell compartment between the 2 strains could be due to differences in genetic background. The DKO strain exhibited fewer IFN-γ-positive T cells in the ELISpot assay following *in vitro* restimulation with FVIII peptide mix, suggesting that the FVIII-specific T-cell response in the DKO strain is partially reduced. Of note, in previous severely immunodeficient HA mouse models, the anti-FVIII T-cell response was abrogated [[30], [31], [32]].

The formation of neutralizing ADAs has previously limited the feasibility of prophylaxis studies in the HA mouse model. This represents a significant limitation, as the majority of novel HA treatments are directed toward improving prophylaxis rather than treating acute bleeds. Half-life extended FVIII products, including the recently developed FVIII molecule efanesoctocog alfa [44], aim to optimize prophylaxis by enabling less frequent dosing and maintaining higher trough levels in people with HA. In contrast, nonfactor replacement therapies are administered subcutaneously and exhibit long half-lives [45]. While these products have shown clinical efficacy in preventing bleeds in people with HA, their comparative efficacy and impact on joint and bone health remain difficult to assess without randomized clinical trials. Even more important, growing evidence suggests that FVIII plays roles beyond coagulation, including long-term effects on synovial microbleeding, regulation of inflammatory responses, and influence on bone remodeling. These functions and their interplay potentially lead to HA-related joint and bone disease, which is difficult to address in people with HA [16,[46], [47], [48]]. Although several HA products show good clinical efficacy in reducing bleeding episodes, it remains unclear why certain long-term complications, such as hemophilic arthropathy, are not adequately prevented [[5], [6], [7]]. Moreover, the commonly used FVIII-deficient HA mouse strain [20,21] proved unsuitable for studying these complications due to its strong immunogenicity to FVIII, limiting its translational relevance [25].

The DKO model thus addresses a critical gap: it supports repeated administration of FVIII products without ADA development, allowing systematic evaluation of long-term primary and secondary prophylactic strategies, including their potential benefits beyond hemostasis—such as joint protection, inflammation control, and tissue remodeling. In normal practice, for future studies focusing on joint and bone health in HA, it is important to establish parallel control groups that are comparable in age, sex, physical activity, and mouse strain to correctly interpret specific characteristics of the strain, such as possible reduced mineral density [49] and background-specific hematological phenotypes.

While the formation of anti-FVIII antibodies was completely absent in DKO mice, we observed variability in antibody titers among SKO mice. A comparison between groups 1 and 2 revealed that the time after the last FVIII injection influenced both the antibody titer and T-cell stimulation results. This suggests that FVIII injection shortly before sampling may have neutralized a part of the antibody and cellular responses in our *ex vivo* assays. Therefore, if the primary objective is to assess antibody titers or cellular responses, FVIII injections should be avoided shortly before sample collection. In our study, the timing of FVIII injection was mainly chosen to allow FVIII PK assessment. The sparse sampling approach applied was effective in estimating PK parameters of a well-characterized rhFVIII concentrate, as evidenced by the low variability observed in the DKO mice. However, for molecules with more complex PK or longer half-lives, additional sampling time points and an increased number of biological replicates may be required to ensure accurate PK assessment.

Although the DKO mouse model presents a valuable tool for long-term studies of HA, several limitations should be acknowledged. First, the model is not suitable for studying emicizumab [50] or anti-human tissue factor pathway inhibitor antibodies, as these agents are species-specific and function only within the context of the coagulation cascade. To enable such studies, further modifications would be required, eg, the injection or transgenic expression of human FIX and FX to study emicizumab. Second, while the DKO model shows a significant reduction in T-cell response to FVIII-derived peptides, this reduction is only partial. A residual response could be relevant in certain contexts, such as gene therapy studies. On the other hand, it may also present an interesting opportunity to study the immunogenicity of modified FVIII molecules without the confounding influence of clearance mediated by ADAs. Third, due to the lack of B cells in the model, we cannot exclude the potential vulnerability of the model to certain pathogens, as reported for the Ighm^-/-^ mouse model [51,52]. Although we did not encounter any significant infections during breeding or experimental procedures, this risk should be considered when planning longer or more complex experimental protocols.

In summary, the DKO mouse model lacks anti-FVIII antibody formation and shows stable, predictable FVIII PK following repeated administration over a 3-week period. This model can serve as a valuable platform for preclinical evaluation of FVIII products, particularly during repeated administration. Moreover, it enables the assessment of the long-term therapeutic effects of FVIII, including impacts on joint and bone health and inflammation, which are critical aspects of modern HA management.

## References

[1] Gitschier J., Wood W.I., Goralka T.M., Wion K.L., Chen E.Y., Eaton D.H. (1984). Characterization of the human factor VIII gene. Nature.

[2] Ling M., Heysen J.P., Duncan E.M., Rodgers S.E., Lloyd J.V. (2011). High incidence of ankle arthropathy in mild and moderate haemophilia A. Thromb Haemost.

[3] Kloosterman F.R., Zwagemaker A.F., Bagot C.N., Beckers E.A.M., Castaman G., Cnossen M.H. (2022). The bleeding phenotype in people with nonsevere hemophilia. Blood Adv.

[4] Rodriguez-Merchan E.C., Valentino L.A. (2019). Increased bone resorption in hemophilia. Blood Rev.

[5] Arvanitakis A., Jepsen C., Andersson N.G., Baghaei F., Astermark J. (2024). Primary prophylaxis implementation and long-term joint outcomes in Swedish haemophilia A patients. Haemophilia.

[6] Scott M.J., Xiang H., Hart D.P., Palmer B., Collins P.W., Stephensen D. (2019). Treatment regimens and outcomes in severe and moderate haemophilia A in the UK: the THUNDER study. Haemophilia.

[7] Rayment R., Chalmers E., Forsyth K., Gooding R., Kelly A.M., Shapiro S. (2020). Guidelines on the use of prophylactic factor replacement for children and adults with haemophilia A and B. Br J Haematol.

[8] Peyvandi F., Mannucci P.M., Garagiola I., El-Beshlawy A., Elalfy M., Ramanan V. (2016). A randomized trial of factor VIII and neutralizing antibodies in hemophilia A. N Engl J Med.

[9] Franchini M., Mannucci P.M. (2011). Inhibitors of propagation of coagulation (factors VIII, IX and XI): a review of current therapeutic practice. Br J Clin Pharmacol.

[10] Meeks S.L., Batsuli G. (2016). Hemophilia and inhibitors: current treatment options and potential new therapeutic approaches. Hematology Am Soc Hematol Educ Program.

[11] Kitazawa T., Igawa T., Sampei Z., Muto A., Kojima T., Soeda T. (2012). A bispecific antibody to factors IXa and X restores factor VIII hemostatic activity in a hemophilia A model. Nat Med.

[12] Kitazawa T., Esaki K., Tachibana T., Ishii S., Soeda T., Muto A. (2017). Factor VIIIa-mimetic cofactor activity of a bispecific antibody to factors IX/IXa and X/Xa, emicizumab, depends on its ability to bridge the antigens. Thromb Haemost.

[13] Chowdary P., Lethagen S., Friedrich U., Brand B., Hay C., Abdul Karim F. (2015). Safety and pharmacokinetics of anti-TFPI antibody (concizumab) in healthy volunteers and patients with hemophilia: a randomized first human dose trial. J Thromb Haemost.

[14] Patel-Hett S., Martin E.J., Mohammed B.M., Rakhe S., Sun P., Barrett J.C. (2019). Marstacimab, a tissue factor pathway inhibitor neutralizing antibody, improves coagulation parameters of *ex vivo* dosed haemophilic blood and plasmas. Haemophilia.

[15] Sehgal A., Barros S., Ivanciu L., Cooley B., Qin J., Racie T. (2015). An RNAi therapeutic targeting antithrombin to rebalance the coagulation system and promote hemostasis in hemophilia. Nat Med.

[16] Olgasi C., Assanelli S., Cucci A., Follenzi A. (2024). Hemostasis and endothelial functionality: the double face of coagulation factors. Haematologica.

[17] Goldscheitter G., Recht M., Sochacki P., Manco-Johnson M., Taylor J.A. (2021). Biomarkers of bone disease in persons with haemophilia. Haemophilia.

[18] Gerstner G., Damiano M.L., Tom A., Worman C., Schultz W., Recht M. (2009). Prevalence and risk factors associated with decreased bone mineral density in patients with haemophilia. Haemophilia.

[19] Yen C.T., Fan M.N., Yang Y.L., Chou S.C., Yu I.S., Lin S.W. (2016). Current animal models of hemophilia: the state of the art. Thromb J.

[20] Bi L., Sarkar R., Naas T., Lawler A.M., Pain J., Shumaker S.L. (1996). Further characterization of factor VIII-deficient mice created by gene targeting: RNA and protein studies. Blood.

[21] Bi L., Lawler A.M., Antonarakis S.E., High K.A., Gearhart J.D., Kazazian H.H. (1995). Targeted disruption of the mouse factor VIII gene produces a model of haemophilia A. Nat Genet.

[22] Rode F., Almholt K., Petersen M., Kreilgaard M., Kjalke M., Karpf D.M. (2018). Preclinical pharmacokinetics and biodistribution of subcutaneously administered glycoPEGylated recombinant factor VIII (N8-GP) and development of a human pharmacokinetic prediction model. J Thromb Haemost.

[23] Pastoft A.E., Lykkesfeldt J., Ezban M., Tranholm M., Whinna H.C., Lauritzen B. (2012). A sensitive venous bleeding model in haemophilia A mice: effects of two recombinant FVIII products (N8 and Advate(®)). Haemophilia.

[24] Vollack-Hesse N., Oleshko O., Werwitzke S., Solecka-Witulska B., Kannicht C., Tiede A. (2021). Recombinant VWF fragments improve bioavailability of subcutaneous factor VIII in hemophilia A mice. Blood.

[25] Reipert B.M., Ahmad R.U., Turecek P.L., Schwarz H.P. (2000). Characterization of antibodies induced by human factor VIII in a murine knockout model of hemophilia A. Thromb Haemost.

[26] Hausl C., Maier E., Schwarz H.P., Ahmad R.U., Turecek P.L., Dorner F. (2002). Long-term persistence of anti-factor VIII antibody-secreting cells in hemophilic mice after treatment with human factor VIII. Thromb Haemost.

[27] Werwitzke S., Vollack N., von Hornung M., Kalippke K., Kutzschbach J., Trummer A. (2015). Deletion or inhibition of Fc gamma receptor 2B (CD32) prevents FVIII-specific activation of memory B cells *in vitro*. Thromb Haemost.

[28] Gu R.L., Liu L., Xie L.Z., Gai W.L., Cao S.S., Meng Z.Y. (2016). Pharmacokinetics and pharmacodynamics of SCT800, a new recombinant FVIII, in hemophilia A mice. Acta Pharmacol Sin.

[29] Kosloski M.P., Pisal D.S., Mager D.E., Balu-Iyer S.V. (2014). Nonlinear pharmacokinetics of factor VIII and its phosphatidylinositol lipidic complex in hemophilia A mice. Biopharm Drug Dispos.

[30] Sternberg A.R., Martos-Rus C., Davidson R.J., Liu X., George L.A. (2024). Pre-clinical evaluation of an enhanced-function factor VIII variant for durable hemophilia A gene therapy in male mice. Nat Commun.

[31] Lin Y., Chang L., Solovey A., Healey J.F., Lollar P., Hebbel R.P. (2002). Use of blood outgrowth endothelial cells for gene therapy for hemophilia A. Blood.

[32] Matsui H., Shibata M., Brown B., Labelle A., Hegadorn C., Andrews C. (2007). *Ex vivo* gene therapy for hemophilia A that enhances safe delivery and sustained *in vivo* factor VIII expression from lentivirally engineered endothelial progenitors. Stem Cells.

[33] Kitamura D., Roes J., Kühn R., Rajewsky K. (1991). A B cell-deficient mouse by targeted disruption of the membrane exon of the immunoglobulin mu chain gene. Nature.

[34] Epstein M.M., Di Rosa F., Jankovic D., Sher A., Matzinger P. (1995). Successful T cell priming in B cell-deficient mice. J Exp Med.

[35] Kannicht C., Ramström M., Kohla G., Tiemeyer M., Casademunt E., Walter O. (2013). Characterisation of the post-translational modifications of a novel, human cell line-derived recombinant human factor VIII. Thromb Res.

[36] Kalippke K., Werwitzke S., von Hornung M., Mischke R., Ganser A., Tiede A. (2009). DNA analysis from stool samples: a fast and reliable method avoiding invasive sampling methods in mouse models of bleeding disorders. Lab Anim.

[37] Percie du Sert N., Ahluwalia A., Alam S., Avey M.T., Baker M., Browne W.J. (2020). Reporting animal research: explanation and elaboration for the ARRIVE guidelines 2.0. PLoS Biol.

[38] EUR-Lex Directive 2010/63/EU of the European Parliament and of the Council of 22 September 2010 on the protection of animals used for scientific purposes. https://eur-lex.europa.eu/eli/dir/2010/63/oj/eng.

[39] Natalini A., Simonetti S., Favaretto G., Peruzzi G., Antonangeli F., Santoni A. (2021). OMIP-079: Cell cycle of CD4^+^ and CD8^+^ naïve/memory T cell subsets, and of Treg cells from mouse spleen. Cytometry A.

[40] Vollack N., Friese J., Bergmann S., Tiede A., Werwitzke S. (2017). CD32 inhibition and high dose of rhFVIII suppress murine FVIII-specific recall response by distinct mechanisms *in vitro*. Thromb Haemost.

[41] Hausl C., Ahmad R.U., Schwarz H.P., Muchitsch E.M., Turecek P.L., Dorner F. (2004). Preventing restimulation of memory B cells in hemophilia A: a potential new strategy for the treatment of antibody-dependent immune disorders. Blood.

[42] LeBien T.W., Tedder T.F. (2008). B lymphocytes: how they develop and function. Blood.

[43] Whitmire J.K., Asano M.S., Kaech S.M., Sarkar S., Hannum L.G., Shlomchik M.J. (2009). Requirement of B cells for generating CD4+ T cell memory. J Immunol.

[44] Demers M., Aleman M.M., Kistanova E., Peters R., Salas J., Seth Chhabra E. (2022). Efanesoctocog alfa elicits functional clot formation that is indistinguishable to that of recombinant factor VIII. J Thromb Haemost.

[45] Franchini M., Mannucci P.M. (2018). Non-factor replacement therapy for haemophilia: a current update. Blood Transfus.

[46] Gualtierotti R., Solimeno L.P., Peyvandi F. (2021). Hemophilic arthropathy: current knowledge and future perspectives. J Thromb Haemost.

[47] Greco T., Polichetti C., Cannella A., La Vergata V., Maccauro G., Perisano C. (2021). Ankle hemophilic arthropathy: literature review. Am J Blood Res.

[48] Leuci A., Dargaud Y. (2023). Blood-induced arthropathy: a major disabling complication of haemophilia. J Clin Med.

[49] Li Y., Toraldo G., Li A., Yang X., Zhang H., Qian W.P. (2007). B cells and T cells are critical for the preservation of bone homeostasis and attainment of peak bone mass *in vivo*. Blood.

[50] Ferrière S., Peyron I., Christophe O.D., Kawecki C., Casari C., Muczynski V. (2020). A hemophilia A mouse model for the *in vivo* assessment of emicizumab function. Blood.

[51] Yang X., Brunham R.C. (1998). Gene knockout B cell-deficient mice demonstrate that B cells play an important role in the initiation of T cell responses to *Chlamydia trachomatis* (mouse pneumonitis) lung infection. J Immunol.

[52] Beland J.L., Sobel R.A., Adler H., Del-Pan N.C., Rimm I.J. (1999). B cell-deficient mice have increased susceptibility to HSV-1 encephalomyelitis and mortality. J Neuroimmunol.

